# Phase I study of nab-paclitaxel, gemcitabine, and bevacizumab in patients with advanced cancers

**DOI:** 10.1038/s41416-018-0068-z

**Published:** 2018-04-26

**Authors:** Shiraj Sen, Shumei Kato, Rishi Agarwal, Sarina Piha-Paul, Kenneth Hess, Daniel Karp, Filip Janku, Siqing Fu, Aung Naing, Shubham Pant, Gerald Falchook, Chad Tang, Xifeng Wu, Yuanqing Ye, Apostolia Tsimberidou, Vivek Subbiah, Razelle Kurzrock, Lauren Byers, Shannon Westin, JoAnn Lim, Stacie Bean, Allison Bass, Ly Nguyen, Funda Meric-Bernstam, David Hong

**Affiliations:** 1 Division of Cancer Medicine, The University of Texas MD Anderson Cancer Center, Houston, TX 77030 USA; 2 Department of Medical Oncology, University of California at San Diego, San Diego, CA 92103 USA; 3 Department of Investigational Cancer Therapeutics (Phase I Clinical Trials Program), The University of Texas MD Anderson Cancer Center, Houston, TX 77030 USA; 4 Department of Biostatistics, The University of Texas MD Anderson Cancer Center, Houston, TX 77030 USA; 5 Sarah Cannon Research Institute at HealthONE, Denver, CO 80218 USA; 6 Department of Epidemiology, The University of Texas MD Anderson Cancer Center, Houston, TX 77030 USA; 7 Department of Thoracic and Head and Neck Medical Oncology, The University of Texas MD Anderson Cancer Center, Houston, TX 77030 USA; 8 Department of Gynecologic Oncology, The University of Texas MD Anderson Cancer Center, Houston, TX 77030 USA

**Keywords:** Chemotherapy, Chemotherapy

## Abstract

**Background:**

We performed a phase I modified 3 + 3 dose escalation study to evaluate the safety and activity of bevacizumab plus gemcitabine and nab-paclitaxel in patients with advanced solid tumours.

**Methods:**

Patients were given fixed dose gemcitabine plus increasing doses of nab-paclitaxel and bevacizumab. Toxicity, response, and association with VEGF polymorphism was analysed.

**Results:**

The study enrolled 110 patients who had undergone a median of 3 prior lines of therapy. The median age was 60 years (range, 17–85 years), and 55 patients (50%) had gemcitabine-refractory disease. We observed 3 dose-limiting toxicities during dose escalation and 3 DLTs in expansion cohorts. Dose escalation to 150 mg/m^2^ nab-paclitaxel and 15 mg/kg bevacizumab with 1000 mg/m^2^ of gemcitabine was well tolerated with no MTD. One patient with gemcitabine-refractory peritoneal papillary carcinoma had a complete response, 13 patients (13%) had partial responses, and 54 patients (52%) had stable disease ≥12 weeks. Exploratory VEGF single nucleotide polymorphism (SNP) analysis was performed on 13 patients.

**Conclusions:**

The combination of gemcitabine, nab-paclitaxel, and bevacizumab is safe, well-tolerated, and has activity in advanced malignancies, including gemcitabine-refractory tumours. Based on this study, the recommended phase 2 dose is gemcitabine 1000 mg/m^2^, nab-paclitaxel 125 mg/m^2^, and bevacizumab 15 mg/kg. VEGF polymorphism data should be evaluated in future bevacizumab-based trials.

## Introduction

Paclitaxel, an anti-microtubule agent approved by the U.S. Food and Drug Administration (FDA), has demonstrated significant activity alone and in combination with other chemotherapeutic or biologic agents in ovarian, breast, and lung cancer and AIDS-related Kaposi sarcoma.^1^ Unfortunately, owing to its hydrophobic nature, paclitaxel must be formulated in a castor oil-derived solvent, which leads to paclitaxel-associated hypersensitivity reactions and myelosuppression that often limit its administration, especially in heavily pretreated patients. Nanoparticle albumin-bound paclitaxel (nab-paclitaxel), a solvent-free formulation of paclitaxel, was developed to reduce such toxicity and decrease infusion time through improved drug delivery.^2^ The U.S. FDA has approved nab-paclitaxel as a single agent for the treatment of metastatic breast cancer^3^ and the combination of nab-paclitaxel plus gemcitabine for the treatment of pancreatic cancer.^4^

In preclinical studies, nab-paclitaxel has demonstrated anti-tumour activity as a single agent and synergistic activity when administered with gemcitabine. Specifically, the addition of nab-paclitaxel increases intratumoural concentrations of gemcitabine.^5^ In a large phase III trial comparing the combination of nab-paclitaxel and gemcitabine with single-agent gemcitabine in patients with metastatic pancreatic cancer, the combination improved the overall response rate from 7% to 22%, improved the progression-free survival (PFS) duration from 3.7 months to 5.5 months, and improved the overall survival duration from 6.7 months to 8.5 months.^4^ The combination of nab-paclitaxel plus gemcitabine has also demonstrated anti-tumour activity and a favorable toxicity profile in early-phase trials in patients with metastatic breast cancer^6^ and thoracic malignancies.^7^

Researchers have proposed several strategies to improve the delivery of nab-paclitaxel plus gemcitabine to tumours. One such strategy involves using anti-angiogenic therapy to target the abnormal tumour vasculature that often prevents drug penetration. It has been suggested that the blockade of vascular endothelial growth factor (VEGF), which stimulates tumour angiogenesis, may improve the delivery of these drugs by inducing morphologic normalisation of the tumour vasculature, improve vessel stability, and reduce protein extravasation and intra-tumoural pressure.^8^ Therefore, to determine whether the addition of VEGF inhibition improves the anti-cancer effects of nab-paclitaxel plus gemcitabine, we performed a phase I dose-escalation trial of the combination of bevacizumab, a monoclonal antibody that inhibits VEGF-A, plus nab-paclitaxel and gemcitabine in patients with advanced cancers. We also assessed the extent to which VEGF polymorphism is associated with survival.

## Methods

### Patients

This study (NCT01113476) was performed at The University of Texas MD Anderson Cancer Center under a protocol approved by MD Anderson’s Institutional Review Board (protocol number 2009–0855). All participants provided written informed consent before entering the study. We enrolled patients with histologically confirmed metastatic or locally advanced solid tumours who were seen at the Clinical Center for Targeted Therapy at MD Anderson between May 2010 and July 2017. Eligible patients had disease that had progressed despite standard therapy or for whom no available therapy would likely increase survival by at least 3 months. Patients were required to be off systemic therapy for at least 4 weeks (or for the time of 5 half-lives, in the case of biologic or targeted agents). Other inclusion criteria included an Eastern Cooperative Oncology Group performance status of 0–2, absolute neutrophil count ≥1000/µl, platelet count ≥50,000/µl, total bilirubin level ≤3 mg/dl, alanine aminotransferase level <5 times the institutional upper limit of normal, creatinine level <3 times the institutional upper limit of normal, and the use of contraception during the study. Patients were excluded if they had a history of myocardial infarction within 6 months prior to starting the treatment, were pregnant or breastfeeding, had uncontrolled hypertension (systolic blood pressure >140 mmHg or diastolic blood pressure >90 mmHg on medication), had any unhealed wounds, and/or had undergone a major surgical procedure and/or had clinically significant, unexplained bleeding within 28 days prior to starting the treatment. Patients with any prior hypersensitivity reaction to bevacizumab, gemcitabine, paclitaxel, and/or nab-paclitaxel were also excluded. Patients were allowed to receive palliative radiation therapy but not allowed to use other standard or investigational anti-cancer agents during the study period.

### Study design and treatment

This study was a non-randomised, open-label, modified 3 + 3 dose escalation study of gemcitabine at a fixed dose of 1000 mg/m^2^ (days 1, 8, and 15), nab-paclitaxel at doses of 50–150 mg/m^2^ (days 1, 8, and 15), and bevacizumab at doses of 5–15 mg/kg (days 1 and 15). The dose escalation design is illustrated in Table 1. The primary objective was to determine the safety, maximum tolerated dose (MTD), and dose limiting toxicities (DLTs) of the drug combination. The secondary objective was to describe the anti-tumour efficacy of the drug combination. Because nab-paclitaxel and bevacizumab were given sequentially in escalating doses, we investigated a variety of dose levels using a modified 3 + 3 study design,^9^ which enabled us to determine multiple potential MTDs of each drug given in combination.

**Table 1:** Dose escalation schedule, number of patients treated, and DLTs

Dose level	Nab-paclitaxel dose, mg/m^2^, days 1, 8, 15	Bevacizumab dose, mg/kg, days 1, 15	Gemcitabine dose, mg/m^2^, days 1, 8, 15	Number of patients treated	Number of patients with DLTs^a^
1	50	5	1000	3	0
2	75	5	1000	6	0
3	50	10	1000	6	1 (thrombocytopenia, dehydration, dysphagia, and dyspnea)
4	100	5	1000	17	2 (1 diarrhoea; 1 fatigue)^b^
5	75	10	1000	16	1 (cellulitis); 2 (GI bleeding)^b^
6	50	15	1000	6	0
7	125	5	1000	7	0
8	100	10	1000	6	0
9	75	15	1000	18	0
10	150	5	1000	6	1 (bacteremia, fatigue, and dehydration)
11	125	10	1000	3	0
12	100	15	1000	3	0
13	150	10	1000	3	0
14	125	15	1000	3	0
15	150	15	1000	3	0

Dosing scheme for patients who received the combination of nab-paclitaxel, gemcitabine, and bevacizumab in a 28-day cycle, as well as number of patients treated at each dose level, and number of patients with DLTs at each dose level.

*IV* intravenous.

^a^All DLTs were grade 3.

^b^Observed in an expansion cohort

An MTD was defined as the dose level below the dose level at which 2 of 6 patients experienced drug-related DLTs in the first cycle. DLTs were any grade 3 or 4 non-haematologic toxicity (except nausea and vomiting responsive to supportive therapy), any grade 4 haematologic toxicity lasting 2 weeks or longer despite supportive care, unless complicated by fever or infection, bleeding symptomatic anaemia, or other symptoms concerning to the treating physician, and grade 4 nausea or vomiting lasting more than 5 days despite maximum therapy. The escalation path was determined by the DLTs that patients at prior dose levels experienced. If 2 patients experienced DLTs at a dose level and the DLTs could both be attributed to either nab-paclitaxel or bevacizumab, all future dose levels with that dose of the drug would be deemed inadmissible. If each of the two DLTs could be attributed to a different drug, 6 additional patients would be enrolled to determine whether the dose level was the one above the MTD. This schema was utilised to allow for the possibility of more than 1 MTD, as may be expected when two drugs at different, escalating dose levels are being investigated. If patients with a particular tumour histology type had a response, the study allowed for an expansion cohort to include a total of 14 patients with that tumour type, who would be treated at the highest current dose level. All such patients would be included in the DLT analysis.

If a patient experienced new grade 3 or higher toxicity, treatment was held until the condition was addressed and recovered to grade 1 or baseline. The treating physicians were then allowed to reduce the dose by up to 50% if the toxicity was attributed to nab-paclitaxel and/or bevacizumab. The patients continued the treatment until they had disease progression or intolerable toxicities; until the treating physicians or patients believed that it was not in the patients’ best interest to continue treatment for any reason; or until patients withdrew consent for any reason.

All patients were evaluated for DLTs during the first 28 days. The patients were evaluated in the clinic every 28 days prior to initiation of each subsequent cycle. Response to therapy was assessed every 2 cycles using Response Evaluation Criteria in Solid Tumours (RECIST) version 1.1.^10^

### VEGF polymorphism analysis

Thirteen patients agreed to have an optional blood draw for VEGF polymorphism analysis. DNA samples were extracted from the blood and genotyping was performed using the custom Infinium Oncoarray Beadchip following the standard Illumina protocol. We identified eight single nucleotide polymorphisms (SNPs) within 10 kb of the VEGF gene. We used the Fisher exact test to analyse the association of these SNPs with treatment response and used the log-rank test to analyse the association of these SNPs with PFS.

## Results

### Patient characteristics

The characteristics of the 110 patients (48 men and 62 women) who received at least 1 dose of nab-paclitaxel, gemcitabine, and bevacizumab are shown in Table 2. The most common tumour types enrolled were ovarian (*n* = 21), pancreatic (*n* = 19), sarcoma (*n* = 10), urothelial (*n* = 7), gastroesophageal junction (*n* = 7), gastric (n = 5), and small cell lung cancer (*n* = 4). The patients’ median age was 60 years (range, 17–85 years). The patients were heavily pretreated with a median of 3 prior lines of therapy; 32 patients (29%) had received 3 prior lines of therapy, 29 (26%) had received 4 prior lines of therapy, and 15 (13%) had received 5 or more prior lines of therapy. Overall, 55 patients (50%) had previously progressed on gemcitabine used either as a single agent or in combination with other anti-cancer agents.

**Table 2:** Baseline demographics

Characteristic	No. of patients (%)
Sex	
Female	62 (55)
Male	48 (45)
Median age at study enrollment (range), years	60 (17–85)
Disease type	
Ovarian cancer	21 (19)
Pancreatic cancer	19 (17)
Sarcoma	10 (9)
Urothelial cancer	7 (6)
Gastroesophageal junction cancer	7 (6)
Gastric cancer	5 (5)
Small cell lung cancer	4 (4)
Other	36 (34)
Median no. of prior lines of therapy (range)	3 (0–9)
≤2	34 (31)
3	32 (29)
4	29 (26)
≥5	15 (13)
Prior gemcitabine	55 (50)
Prior bevacizumab	27 (25)

Baseline demographics of all 110 patients treated on phase 1 trial with nab-paclitaxel, gemcitabine, and bevacizumab. Sex, age, disease type, number of lines of therapy, and number of patients with prior gemcitabine and prior bevacizumab use are all listed.

Note: All data are no. of patients (%) unless otherwise indicated

Of the 110 patients who received at least 1 dose of study drug, 4 did not complete the first treatment cycle, were replaced from the study and deemed not evaluable for toxicity or response. (Of these patients, 2 elected to go to hospice after receiving 1 dose of the study drug, and the other 2 received 1 dose of the drug and were admitted to the hospital for failure to thrive secondary to advanced cancer.) Of the remaining 106 patients, 12 were evaluable for toxicity but not response, as they withdrew consent (*n* = 5), died prior to first restaging (*n* = 4), or were on dose level 15 and had not yet completed first restaging (*n* = 3). The remaining 94 patients were evaluable for response.

### Safety

Among the 106 patients evaluable for toxicity, three DLTs were observed during dose escalation: one at dose level 3 with nab-paclitaxel 50 mg/m^2^ and bevacizumab 10 mg/kg (grade 3 thrombocytopenia, dehydration, dysphagia, and dyspnea); one at dose level 5 with nab-paclitaxel 75 mg/m^2^ and bevacizumab 10 mg/kg (grade 3 cellulitis); and one at dose level 10 with nab-paclitaxel 150 mg/m^2^ and bevacizumab 5 mg/kg (grade 3 bacteremia, fatigue, and dehydration). Two DLTs (one grade 3 diarrhoea and one grade 3 fatigue) were observed among the 14 patients in the dose level 4 expansion cohort (14%) receiving nab-paclitaxel 100 mg/m^2^ and bevacizumab 5 mg/kg. Two DLTs (both grade 3 gastrointestinal bleeding) were observed among the 13 patients in the dose level 5 expansion cohort (15%) with nab-paclitaxel 75 mg/m^2^ and bevacizumab 10 mg/kg (Table 1). Dose escalation up to nab-paclitaxel 150 mg/m^2^ and bevacizumab 15 mg/kg was well tolerated, with no MTD achieved.

All grade 3 and 4 toxicities experienced during dose escalation and dose expansion are listed in Table 3. The most common grade 3 toxicity was neutropenia, which occurred in 19 patients (18%), followed by fatigue in 8 patients (8%), infection in 7 patients (7%), thrombocytopenia in 6 patients (6%), and nausea in 6 patients (6%). Grade 4 haematologic toxicities included neutropenia in 7 patients (7%) and thrombocytopenia in 3 patients (3%). No grade 4 non-haematologic toxicities were observed. Sixty-two patients (58%) required at least 1 dose of a drug to be held, and 29 patients (27%) required dose reductions. Owing to toxicity, 22 patients (21%) required gemcitabine dose reductions, 20 patients (19%) required nab-paclitaxel dose reductions, and 4 patients (4%) required bevacizumab dose reductions.

**Table 3:** All grade 3 and 4 toxicities

Toxicity	Grade	No. of patients (%)
Neutropenia	3	19 (18)
	4	7 (6)
Fatigue	3	8 (7)
Thrombocytopenia	4	3 (3)
	3	6 (5)
Infection	3	7 (6)
Nausea	3	6 (5)
Diarrhoea	3	2 (2)
Lymphedema	3	1 (1)
Dysphagia	3	1 (1)
Dyspnea	3	2 (2)
GI bleed	3	3 (3)
	2	1 (1)
Anaemia	3	4 (4)
Fever	3	3 (3)
	2	1 (1)
Hypotension	3	2 (2)
Abdominal pain	3	2 (2)
	2	1 (1)
Dysphagia	3	2 (2)

All grade 3 and 4 toxicities as well as number of patients experiencing each toxicity while being treated with nab-paclitaxel, gemcitabine, and bevacizumab.

*GI* gastrointestinal

### Response

Of the 106 patients who completed the first treatment cycle, 12 were not evaluable for response. Of these 12 patients, 5 were on the protocol through the first cycle (DLT window) and were evaluable for toxicity but withdrew consent prior to the first restaging; 4 completed the first cycle but died prior to the first restaging (1 from septic shock, 1 from pneumonia, and 2 from unknown etiologies); and 3 had yet to complete restaging. The deaths were not considered to be due to the study drugs. Of the remaining 94 patients who underwent restaging and were evaluable for response, 50 (53%) had objective tumour volume reduction as best response. Overall, 1 patient with metastatic primary peritoneal papillary carcinoma treated on dose level 5 with nab-paclitaxel 50 mg/m^2^ and bevacizumab 10 mg/kg had a complete response. This patient’s disease had been refractory to gemcitabine plus carboplatin as well as a docetaxel plus bevacizumab. Of the 14 patients (14%) who had a partial response (PR), 6 had disease that had been refractory to gemcitabine-based therapy.

**Fig. 1:**
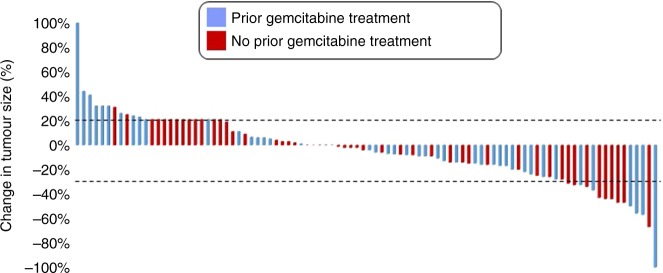
Waterfall plot depicting best response as a percent change in target lesion size in all evaluable patients. Patients previously treated with gemcitabine are indicated with blue

The treatment responses of the 94 patients evaluable for response are shown in Fig. 1. Fourteen patients (15%)—1 with ampulla of vater adenocarcinoma, 1 with GE junction adenocarcinoma, 1 with pancreatic adenocarcinoma, 2 with squamous cell carcinoma of the oesophagus, 2 with B cell lymphoma, 4 with ovarian adenocarcinoma, 2 with small cell lung cancer, and 1 with small cell carcinoma of the bladder—had PRs. Among these 14 patients who achieved a PR, 1 of the 2 patients with B cell lymphoma, 3 of the 4 patients with ovarian adenocarcinoma, and the 1 patient with pancreatic adenocarcinoma had gemcitabine-refractory disease. Twenty-five patients (27%) had disease progression at first restaging. Two patients with leiomyosarcoma and 1 patient with ovarian adenocarcinoma remain on protocol with continued clinical response after 5, 7, and 7 months, respectively.

**Fig. 2:**
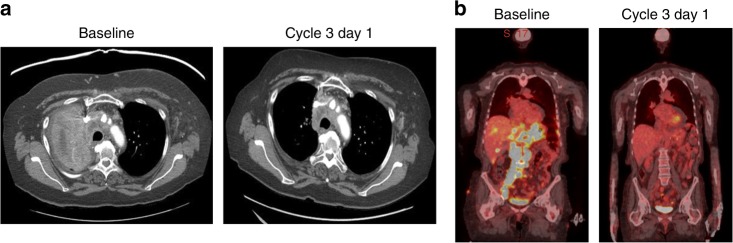
Notable responses to gemcitabine, nab-paclitaxel, and bevacizumab. Representative restaging images of two patients who had notable responses to therapy. **a** A patient with small cell lung cancer treated on dose level 9 who had a partial response after disease progression on first-line carboplatin plus etoposide and second-line topotecan. **b** A patient with B-cell lymphoma treated on dose level 5 who had a partial response after disease progression on R-CHOP (rituximab, cyclophosphamide, doxorubicin, vincristine, and prednisone), DHAP (rituximab, cisplatin, cytarabine, and dexamethasone), BR (bendamustine and rituximab), and an experimental interleukin-1 antagonist

Notable responses were seen in 4 subsets of patients—gastroesophageal cancers, pancreatic cancer, small cell cancers, and ovarian cancer. Of the 17 patients with gastroesophageal cancers, 3 had PRs, 10 had stable disease (SD), and 4 had disease progression as best response. Both the patients with squamous cell carcinoma of the oesophagus had PRs with 44% and 47% tumour shrinkage, respectively, as best response. Of the 15 patients with pancreatic cancer, 1 had a PR with 57% tumour shrinkage, 10 (67%) had SD (9 of these patients had tumour shrinkage as best response), and 4 (27%) had disease progression before or at first restaging. Among the 6 patients with small cell cancers—4 with small cell lung cancer, 1 with small cell prostate cancer, and 1 with small cell bladder cancer—only 1 of the small cell lung cancer patient had disease progression as best response, and the other 5 patients had tumour shrinkage of at least 25% as best response. Finally, of the 17 patients with ovarian cancer and measurable disease, 4 (24%) had PR as best response, 9 patients (53%) had SD as best response, and 4 patients (24%) had progressive disease (PD) as best response. Two additional ovarian cancer patients who had no measurable disease at the time of trial enrollment had SD for more than 6 months before having disease progression. Of the 19 evaluable patients with ovarian cancer, 8 (44%) had serous carcinomas, and each of these patients had tumour shrinkage of at least 20% as best response, representing 4 PRs and 4 SDs. Of the 8 patients with tumour shrinkage, 6 (75%) previously had gemcitabine-refractory disease. Representative images of patients with notable responses are shown in Fig. 2.

### VEGF polymorphism analysis

Response rates and PFS duration of the 13 patients that agreed to optional VEGF polymorphism data collection were analysed. Eight of the nine VEGF SNPs analysed demonstrated no statistical association between SNP genotype and PFS. The PFS duration of the 2 patients with the GA genotype in the rs6900017 VEGF SNP (84 days, 95% CI = 84–not reached) was significantly shorter than that of the 11 patients with the GG genotype in the SNP (308 days, 95% CI = 91–392, *p* = 0.02).

## Discussion

Gemcitabine remains a backbone for the treatment of pancreatic, ovarian, lung, and breast cancer. The addition of nab-paclitaxel to gemcitabine is well tolerated in thoracic and breast malignancies^7, 11^ and improves PFS and overall survival in pancreatic cancer.^4^ The MTD of gemcitabine and nab-paclitaxel in advanced pancreatic cancer was previously found to be gemcitabine 1000 mg/m^2^ and nab-paclitaxel 125 mg/m^2^.^12^ The results of this phase I trial identify that the addition of bevacizumab to gemcitabine and nab-paclitaxel is safe, well tolerated, and has clinical activity in patients with advanced cancers. The MTD was not reached, and dose escalation to the highest prespecified doses of gemcitabine (1000 mg/m^2^), nab-paclitaxel (150 mg/m^2^), and bevacizumab (15 mg/kg) was achieved.

In order to promote generalisability of our results and include representative patients with advanced cancer on this trial, broad eligibility criteria were applied and allowed all patients with platelet counts greater than 50 × 10^3^/µl, bilirubin levels less than 3.0 mg/dl, and creatinine levels less than 3 times the upper limit of normal. Many of these patients have traditionally been excluded from gemcitabine-based trials and further analysis is ongoing to evaluate whether broadening certain eligibility criteria may have contributed to the presence of toxicities in a non-dose dependent fashion in this and in other trials. In the present study, DLTs included grade 3 sepsis, bleeding, fatigue, and diarrhoea. Toxicities were neither dose dependent nor associated with prior gemcitabine use, prior nab-paclitaxel use, age, or performance status. Sepsis was not an unexpected DLT as it was also seen in early-phase studies combining gemcitabine and nab-paclitaxel.^12^ The incidences of grade 3 fatigue and diarrhoea and grade 2 peripheral neuropathy—non-haematologic toxicities known to occur in select patients treated with gemcitabine and nab-paclitaxel^12^—were lower in this trial than in previous studies, likely owing to improvements in supportive care over the last decade.

Interestingly, of the 14 patients who had grade 1 or 2 peripheral neuropathy, 13 experienced tumour shrinkage. In the randomised phase III MPACT trial, a subset analysis demonstrated an association between peripheral neuropathy and survival in patients with metastatic pancreatic adenocarcinoma treated with gemcitabine and nab-paclitaxel.^13^ Our data suggest a similar association in non–pancreatic cancer patients treated with this combination of drugs. Moreover, our data highlight the importance of recognising and optimally managing peripheral neuropathy through dose reduction and pharmacologic intervention to enable patients to continue therapy and potentially derive benefit from it.

At dose level 5 (10 mg/kg bevacizumab), 2 patients had DLTs in the form of grade 3 bleeding. One of these patients had recently started therapeutic doses of enoxaparin sodium for a venous thromboembolism and had grade 1 thrombocytopenia, which likely triggered bleeding from his small bowel adenocarcinoma on cycle 1 day 14. The second patient, who had metastatic pancreatic cancer that was refractory to gemcitabine plus cisplatin as well as capecitabine plus irinotecan, had been on the trial for 5 months with a sustained PR (57% tumour shrinkage from baseline). This patient had recently undergone aortic valvuloplasty for severe aortic stenosis and was taking aspirin and clopidogrel, which likely increased the risk for bleeding. These findings underscore the need to constantly monitor patients treated with bevacizumab for gastrointestinal hemorrhage risk, especially those concurrently receiving anti-platelet and/or anti-coagulation therapy. It has been hypothesised in preclinical studies that the enhanced hemorrhagic toxicity from this combination is a result of nab-paclitaxel upregulation of VEGF-A production, but the combination could elicit complete responses despite the absence of such responses with either agent alone.^14^

Our trial also demonstrates that the combination of gemcitabine, nab-paclitaxel, and bevacizumab has activity even in previously gemcitabine-refractory cancers. The overall response rate of the 94 patients who were evaluated for response was 16%. In particular, 1 patient whose metastatic peritoneal papillary carcinoma had been refractory to gemcitabine and to docetaxel plus bevacizumab had a complete response. In the present study, patients with pancreatic cancer, gastroesophageal cancer, small cell cancer, and ovarian cancer had clinical responses to the combination therapy. Further data to support the combination of nab-paclitaxel, bevacizumab, and gemcitabine has previously been published in a case report that noted a 3-year complete remission in a patient with metastatic triple-negative breast cancer.^15^ Similarly, the combination of hepatic arterial infusion of nab-paclitaxel plus systemic gemcitabine and bevacizumab has had activity in patients with advanced cancers and liver metastases.^16^

Bevacizumab has been added to other gemcitabine-based combination therapies for pancreatic cancer but has not improved survival.^17, 18^ However, given the favorable safety profile associated with the addition of bevacizumab to gemcitabine and nab-paclitaxel in the present trial, further investigation of this combination in a phase 2 study in patients with gastroesophageal, ovarian, or small cell cancers is being considered. Paclitaxel-based regimens are already approved by the U.S. FDA for use in gastroesophageal cancers,^19^ and nab-paclitaxel has been found to be non-inferior to paclitaxel in patients with advanced gastric cancer.^20^ Moreover, early-phase trial data suggest that the addition of front-line bevacizumab is well tolerated and improves outcomes in patients with advanced gastroesophageal adenocarcinomas.^21^ Similarly, bevacizumab and paclitaxel are approved by the FDA for platinum-sensitive ovarian cancers,^22^ and nab-paclitaxel–based regimens have demonstrated activity against small cell lung cancer.^23^ Such data underscore the need to identify a biomarker to predict response to this regimen in order to determine whether it may be clinically relevant to consider in these cancers.

A previous study suggests that VEGF polymorphisms predict response to bevacizumab treatment.^24^ In the present study, polymorphisms in the rs6900017 VEGF SNP were associated with statistically significant differences in PFS. The PFS duration of patients with the GA genotype was significantly shorter than that of patients with the GG genotype. However, the optional, exploratory analysis included only 13 patients, and further investigation of the association of response to bevacizumab-based regimens and VEGF is warranted in order to determine efficacy. Others have hypothesised that polymorphisms in IL8, eNOS,^25^ and autophagy-related genes^26^ predict bevacizumab-based therapy response and toxicity, further suggesting that genomic signatures may be used to help oncologists individualise cancer therapy.

As is the case with all non-randomised phase I trials, patient selection in the present study may have affected the outcomes. Further investigation of the combination of gemcitabine, nab-paclitaxel, and bevacizumab in patients with gastroesophageal, small cell, and ovarian cancers is warranted. The combination of gemcitabine and nab-paclitaxel is one of the options for treatment of patients with advanced pancreatic cancer. This study demonstrates that the combination of gemcitabine, nab-paclitaxel, and bevacizumab can shrink even gemcitabine-refractory pancreatic tumours. Further studies to identify biomarkers that predict response to bevacizumab-based therapy are needed.

## Availability of data and material

All data supporting the results reported in the article were obtained with permission from the MD Anderson’s Institutional Review Board and are stored in the Investigational Cancer Therapeutics department.
